# An Easy and Sensitive Method to Profile the Antibody Specificities of HLA–specific Memory B Cells

**DOI:** 10.1097/TP.0000000000002516

**Published:** 2019-03-22

**Authors:** Gonca E. Karahan, Juliette Krop, Caroline Wehmeier, Yvonne J.H. de Vaal, Janneke Langerak–Langerak, Dave L. Roelen, Neubury M. Lardy, Frederike J. Bemelman, Ineke J.M. ten Berge, Marlies E.J. Reinders, Cees van Kooten, Frans H.J. Claas, Sebastiaan Heidt

**Affiliations:** 1 Department of Immunohematology and Blood Transfusion, Leiden University Medical Center, Leiden, The Netherlands.; 2 Department of Immunogenetics, Sanquin Diagnostic Services, Amsterdam, The Netherlands.; 3 Renal Transplant Unit, Department of Nephrology, Academic Medical Center, Amsterdam, The Netherlands.; 4 Department of Internal Medicine (Nephrology), Leiden University Medical Center, Leiden, The Netherlands.

## Abstract

**Background.:**

Pretransplant immunological risk assessment is currently based on donor–specific HLA antibodies in serum. Despite being an excellent source for antibodies produced by bone marrow–residing plasma cells, serum analysis does not provide information on the memory B–cell compartment. Although B–cell culture supernatants can be used to detect memory B cell–derived HLA antibodies, low IgG concentrations can preclude detectability of HLA antibodies in luminex single–antigen bead (SAB) assays.

**Methods.:**

Culture supernatants of polyclonally activated B cells from alloantigen exposed (n = 13) or nonexposed (n = 10) individuals were either concentrated 10–fold, or IgG was isolated by using a protein G affinity purification method to increase the IgG concentration. These processed culture supernatants, as well as paired serum samples were tested for the presence of HLA antibodies using luminex SAB analysis.

**Results.:**

In immunized individuals, 64% were found to have HLA–specific B–cell memory in concentrated supernatants, whereas 82% showed HLA–specific B–cell memory when IgG isolated supernatants were used for HLA antibody detection. IgG–isolated supernatants showed higher mean fluorescence intensity values compared with concentrated supernatants without increased background. In some individuals, HLA–specific B–cell memory was detected in the absence of accompanying serum antibody specificities.

**Conclusions.:**

We developed a novel, highly sensitive method to assess the HLA–specific memory B–cell compartment using luminex SAB technology. This assay allows direct comparison to the serum compartment and may therefore provide a more complete picture of the humoral alloimmune response in patients with a history of alloantigen exposure.

## Abstract 2

Supplemental Digital Content is available in the text.

Transplant recipients with preexisting or de novo donor–specific HLA antibodies (DSA) are at increased risk for developing antibody–mediated rejection which may significantly impact allograft longevity.^1-3^ In routine clinical practice, immunological risk assessment for transplant candidates is based on the presence of DSA in serum samples. Because circulating antibodies are mainly produced by bone marrow–residing plasma cells, this risk assessment does not provide information on the memory B–cell compartment. However, in patients with a history of alloantigen exposure, circulating memory B cells may also contribute to HLA antibody production by differentiating into antibody–secreting cells (ASC) upon antigen rechallenge or bystander activation.^4,5^ Until now, several methods have been developed to detect HLA–specific memory B cells.^6-13^ Among these, screening for HLA antibody specificities using luminex single–antigen bead (SAB) assays in culture supernatants of in vitro activated B cells is a straightforward method to determine the presence and specificity of circulating HLA–specific memory B cells.^7,11,14^ By using this method, Han et al^7^ showed that a proportion of memory B cell–derived HLA antibody specificities was absent in serum samples from alloantigen exposed individuals. Subsequently, Snanoudj et al^14^ reported that HLA antibody specificities detected in culture supernatants generally had a more restricted specificity pattern compared with serum antibodies; however, some specificities found in the memory B–cell compartment were absent in serum.

Although B–cell supernatant analysis enables direct comparison of HLA antibody specificities to those in serum, it may suffer from the relatively low IgG concentrations in the supernatants that can be below the detection limit of SAB assays. Here, we introduce a novel method consisting of potent nonbiased activation of memory B cells combined with an efficient method of isolating IgG from culture supernatants to assess the HLA–specific memory B–cell compartment with high sensitivity using luminex SAB technology.

## MATERIALS AND METHODS

### Cells

Peripheral blood and serum samples from patients awaiting repeat transplantation (re–tx) (n = 11), multiparous women (n = 2), and individuals who had never been exposed to alloantigens (n = 10) were obtained with informed consent under guidelines issued by the medical ethics committee of Leiden University Medical Center (Leiden, the Netherlands) and Academic Medical Center (Amsterdam, the Netherlands). Peripheral blood mononuclear cells (PBMC) were isolated using Ficoll–Hypaque density gradient centrifugation and kept frozen in liquid nitrogen until further use.

### Polyclonal Activation of B Cells

Polyclonal B–cell activation was carried out by stimulating 2 × 10^6^ PBMC/well in a total volume of 2 mL/well in 24–well plates with an activation cocktail consisting of 2.5 μg/mL Toll–like receptor 7/8 agonist (resiquimod [R848]; Sigma–Aldrich, St. Louis, MO) and 1000 IU/mL IL–2 (Proleukin, Novartis, the Netherlands).^15^ Peripheral blood mononuclear cell cultures were carried out in Iscove’s modified Dulbecca’s medium (Gibco Invitrogen, Paisley, UK) containing 10% fetal bovine serum (Gibco Invitrogen) and 100 U/mL penicillin with 100 μg/ml streptomycin (Gibco Invitrogen). Supernatants harvested at day 6 (d6) or day 10 (d10) were kept at −20°C until further use. Flow cytometry was performed before (d0) and after (d6, d10, and d14) polyclonal activation according to standard protocols using the following antibodies (clone): CD19 (A07770), CD27 (1A4CD27), CD38 (LS198–4–3) and CD45 (J33) (all from Beckman Coulter, Woerden, the Netherlands).

### Supernatant Preparation for HLA Antibody Testing

Culture supernatants were either concentrated 10–fold using ultra centrifugal filters (Amicon Ultracel 50K; Millipore, Ireland), or IgG was isolated by using a protein G affinity purification method. Briefly, 8–mL culture supernatant was loaded onto 200 μL of protein G containing resin (Amicon ProAffinity Concentration Kit; Millipore, Ireland) and gently mixed on a roller bench for 1 hour at room temperature. After a wash step, IgG was eluted, neutralized and further concentrated using ultra centrifugal filters in the same purification device (Amicon ProPurification Device; Millipore). At the end of the procedure, 25 to 35 μL of eluates containing isolated IgG were obtained from 8 mL of start volume. Processed and neat culture supernatants were frozen at −20°C before testing for the presence of HLA antibodies.

### HLA Antibody Detection

All serum samples, as well as processed and neat culture supernatants were tested by ELISA for IgM and IgG concentrations,^16^ in addition to HLA class I and class II antibody detection using Lifecodes SAB kits (LSA; Immucor Transplant Diagnostics, Stamford, CT), based on a previously described protocol using 75% of reagents.^17^ Serum samples were treated with ethylenediaminetetraacetic acid (EDTA) before HLA antibody testing. Data was analyzed using MATCHIT! antibody software version 1.3.0 (Immucor), as provided by the manufacturer. Background corrected mean fluorescence intensity (MFI) values (BCM), BCR (BCM divided by the raw MFI of the lowest ranked bead for a locus) and AD–BCR (antigen density corrected BCR values) were calculated by the software. Bead positivity was defined according to manufacturer’s instructions: for HLA class I: BCM > 1500, BCR > 3, AD–BCR > 4 (minimum, 2 of 3 parameters positive) and for HLA class II: BCM > 1500, BCR > 4, AD–BCR >5 (minimum, 2 of 3 parameters positive). Comparisons for HLA antibody specificities were made between serum and PBMC samples either drawn at the same date or at a median interval of 1 month.

### HLA Typing

All individuals in the study cohort were HLA typed by next–generation sequencing (NGS) for HLA–A, B, C, DRB1, DRB3/4/5, DQB1, DQA1, DPB1, and DPA1 loci on Illumina platform (Illumina, San Diego, CA). Genomic DNA was prepared from peripheral blood samples using kits for automated bead–based DNA isolation (Chemagen, Perkin Elmer, Germany). Locus specific amplification was performed using NGSGo–AmpX reagents (GenDx, Utrecht, the Netherlands). After target generation, library preparation for Illumina platform was performed using NGSgo–LibrX/Indx kits (GenDx). Thereafter, clonal amplification and sequencing steps were carried on Illumina MiniSeq. Data analysis was performed using NGSengine software (GenDx).

### Flow Cytometric Crossmatch

Flow cytometric crossmatches (FCXMs) were performed with selected eluates prepared from 10–day stimulated B–cell cultures using standard FCXM protocols. Briefly, HLA typed PBMC (0.5 × 10^6^) from healthy individuals were incubated with 25 μL of IgG isolated supernatants or specific control sera for 30 minutes at room temperature. After washing three times with phosphate–buffered saline, samples were stained with mouse antihuman CD3–phycoerythrin (clone:SK7), CD19–allophycocyanin (clone: HIB19, both from BD Biosciences, Breda, the Netherlands) and rabbit anti–human IgG F(ab´)_2_–fluorescein isothiocyanate (Dako, Leiden, the Netherlands) for 30 minutes, in the dark at 4°C. After 2 wash steps with phosphate–buffered saline, cells were fixed in 1% paraformaldehyde and acquired on an Accuri C6 flow cytometer (BD Biosciences). T–cell crossmatches were considered positive if the ratio of the MFI of the sample to the MFI of the negative control serum was greater than 1.6. For B–cell crossmatches, an MFI ratio above 2.6 was considered positive.

### Statistical Analysis

Comparisons within groups were performed using Wilcoxon rank analysis. The Mann–Whitney *U* test was used for comparisons between different groups. *P* value <0.05 was considered statistically significant.

## RESULTS

### Ten–day Polyclonal B–cell Activation Results in Maximum IgG Accumulation

**FIGURE 1.**
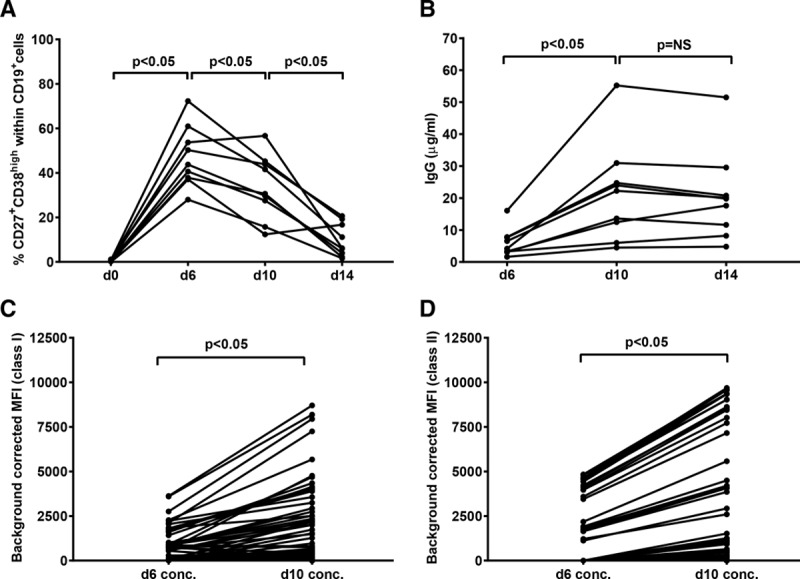
Ten-day polyclonal activation results in maximum IgG accumulation and higher MFI values compared to 6 days of activation. A, Percentages of ASCs (CD19^+^CD27^+^CD38^high^) before (d0) and at d6, d10, and d14 of polyclonal activation (n = 9 [re-tx n = 3, multiparous n = 1 and alloantigen nonexposed n = 5]). B, IgG concentrations at d6, d10, and d14 of polyclonal activation (n = 9). C and D, Background corrected MFI values of all beads for HLA class I and class II measured in d6 and d10 concentrated culture supernatants (n = 13 [re-tx n = 11 and multiparous n = 2]). Each dot represents BCM value of one bead. ASC, antibody-secreting cells; BCM, background corrected MFI values; MFI, mean fluorescence intensity; re-tx, repeat transplantation; d0, day 0; d6, day 6; d10, day 10; d14, day 14.

From our previous work on memory B–cell detection,^12^ we know that culturing PBMC samples (containing a median of 4% CD19^+^ B cells) using a polyclonal activation cocktail consisting of R848 and IL–2 results in selective proliferation of B cells with a median 45% of lymphocytes being CD19^+^ B cells at day 6. This polyclonal activation induces differentiation of B cells into ASC as evident by IgM and IgG production in addition to the acquisition of the characteristic ASC phenotype (CD19^+^CD27^+^CD38^high^). Moreover, we have previously shown that current method of polyclonal activation triggers IgG production by existing memory B cells in the absence of an IgG response by naive B cells, suggesting that naive B cells do not differentiate into memory B cells under these conditions.^12^ Because we hypothesized that HLA antibody detection sensitivity can be increased when higher IgG concentrations in culture supernatants are achieved, we first aimed at defining the culture time with the highest IgG yield in supernatants. To this end, we polyclonally activated PBMC samples using R848 and IL–2 for 6, 10, and 14 days and measured the percentage of ASC at each time point to gain insight into the polyclonal activation of B cells. In addition, we collected supernatants at d6, d10, and d14 to measure total IgG concentrations. Upon polyclonal activation, the median percentage of ASC (CD27^+^CD38^high^ within CD19^+^ B cells) significantly increased from 0.1% (range, 0%–1%) before activation (d0) to 43.8% at d6 (range, 28%–72.3%, *P* < 0.05) followed by a gradual decrease to 30.6% (range, 12.4%–56.7%) at d10 and to 6.2% at d14 (range, 1.6%–20.6%) (*P* < 0.05) (Figure 1A and **Figure S1, SDC,** http://links.lww.com/TP/B653). Notably, CD19^+^ CD27^+^CD38^high^ cells from both patients and healthy individuals behaved similarly in response to the stimuli and the decrease in the percentage of ASC observed from d6 onward was likely due to cell death. Furthermore, IgG concentration in culture supernatants increased significantly from a median of 4.2 μg/mL (range, 1.6–16 μg/mL) at d6 to 22.2 μg/mL (range, 4.5–55.2 μg/mL) at d10 (*P* < 0.05), after which no further increase was found at d14 (median, 19.8 μg/mL; range, 4.8–51.5 μg/mL; *P* = NS) (Figure 1B). Because culturing beyond 10 days did not increase IgG levels, subsequent experiments were performed using only 6– and 10–day stimulated samples. Next, we 10–fold concentrated d6 and d10 culture supernatants to further increase the IgG concentrations and tested for the presence of HLA antibodies using luminex SAB assay (n = 13). Prolonging the culture time from 6 to 10 days led to a significant increase in MFI values for 4.6% class I and 4.8% of class II HLA–coated beads in 10–fold concentrated culture supernatants (*P* < 0.05) (Figure 1C and D), suggesting that higher IgG concentrations in culture supernatants indeed lead to higher MFI values in luminex SAB assay.

### IgG Isolation From Culture Supernatants Restores Physiological IgM/IgG Ratios

**FIGURE 2.**
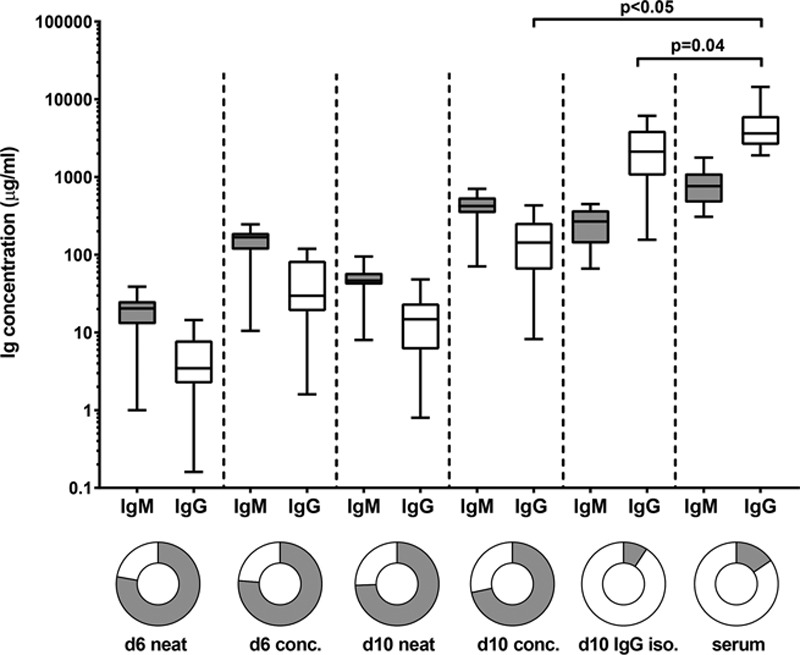
IgG isolation from culture supernatants results in IgM/IgG ratios similar to serum samples. IgM and IgG concentrations in neat (d6 and d10), concentrated (d6 and d10) and IgG isolated (d10) supernatants as well as serum samples (n = 16 [re-tx n = 9, multiparous n = 2 and alloantigen nonexposed n = 5]). Gray bars represent IgM, white bars represent IgG. d6 conc, day 6 concentrated; d10 conc, day 10 concentrated; d10 IgG iso, day 10 IgG isolated; re-tx, repeat transplantation.

Having shown that 10–fold concentration of d10 culture supernatants were superior to d6 for detecting HLA antibodies, we next introduced an IgG isolation step to our d10 supernatant processing protocol to further increase IgG concentrations and to reduce the potentially interfering high levels of IgM (Figure 2). To this aim, we tested IgM and IgG concentrations in culture supernatants and paired serum samples (n = 16).

Whereas 10–fold concentration of d6 and d10 culture supernatants led to median IgG concentrations of 30 and 143 μg/mL, respectively, a median IgG concentration of 2119 μg/mL was achieved by isolating IgG from d10 culture supernatants, which reached IgG concentration found in serum samples (Figure 2). In addition, IgG isolation resulted in an IgM and IgG composition (IgM/IgG, 0.1; IgM mean ± SD, 265 ± 119 μg/mL and IgG mean ± SD, 2592 ± 1764 μg/mL) comparable to serum (IgM/IgG, 0.2; IgM mean ± SD, 849 ± 439 μg/mL and IgG mean ± SD, 4643 ± 3108 μg/mL) (*P* = NS). Although serum samples were similar in their IgM/IgG ratios between patients and healthy individuals, IgM/IgG ratios in culture supernatants were significantly higher in the patient group in comparison to healthy individuals (data not shown). However, despite lower IgG concentrations in d10 IgG isolated culture supernatants of repeat transplant candidates (n = 9; median, 1544 μg/mL, range, 156–2175) compared with healthy individuals (n = 7; median, 3986 μg/mL; range, 3381–6130) (*P* < 0.05), it was still possible to detect HLA–specific memory as well as memory to tetanus–toxoid (**Figure S2, SDC,** http://links.lww.com/TP/B653). Altogether, these results suggested that d10 IgG isolated culture supernatants may be used to fairly compare HLA antibody profiles of the memory B–cell compartment to those found in serum samples.

### IgG Isolation Results in Higher MFI Values Without an Increase in Background

**FIGURE 3.**
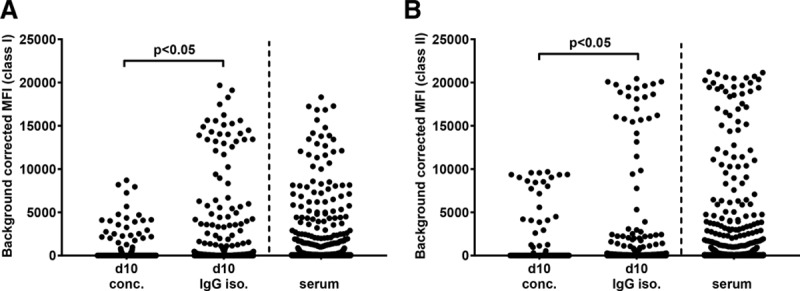
IgG isolation results in higher MFI values in culture supernatants when compared with mere concentration of supernatants. Background corrected MFI values of all HLA class I (A) and class II (B) beads (n = 11 [re-tx n = 9 and multiparous n = 2]). MFI, mean fluorescence intensity; re-tx, repeat transplantation; d10 conc., day 10 concentrated; d10 IgG iso., day 10 IgG isolated.

To determine whether IgG isolation will lead to higher MFI values in comparison to mere concentration, we tested the detectability of HLA antibodies in the different culture supernatant conditions using luminex SAB assay (n = 11). As shown in Figure 3A and B, isolation of IgG from culture supernatants (d10 IgG isolated) resulted in higher MFI values of 27.1% of class I and 43.3% of class II beads in comparison to d10 concentrated samples. Despite this increase in MFI values in d10 IgG–isolated supernatants, median MFI values for self HLA-coated beads were 0 for both class I (range, 0–205) and class II (range, 0–257) (**Figure S3, SDC,** http://links.lww.com/TP/B653). Negative and positive control beads had MFI values <221 and < 11 000, respectively. These results show that increasing IgG concentrations by isolating IgG from culture supernatants leads to higher MFI values only in specific beads without increasing the background.

### IgG Isolation Results in Detection of a Broader Range of HLA Antibody Specificities

Next, we compared the number of positively assigned HLA antibody specificities in d10 culture supernatants to the serum specificities in alloantigen exposed individuals (n = 11) with serum class I and/or class II HLA antibodies (class I only, n = 2; class II only, n = 4; class I + II, n = 5).

**FIGURE 4.**
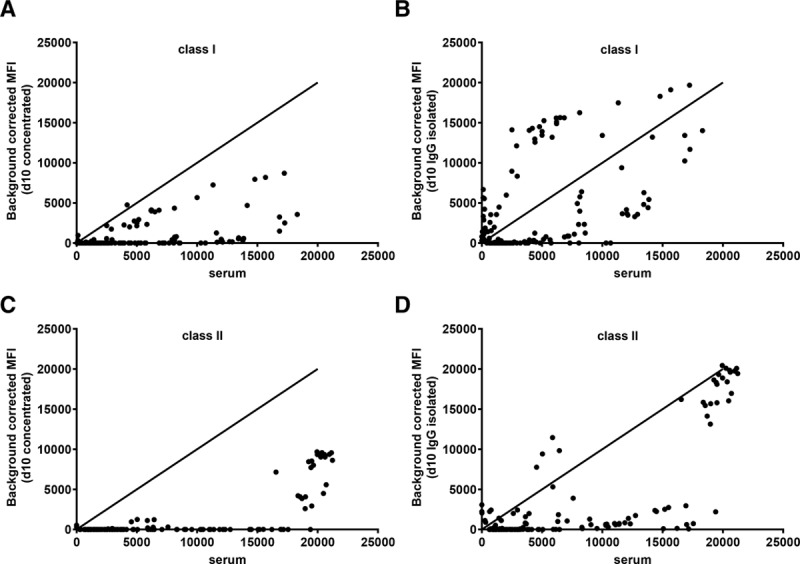
Broader range of HLA antibody specificities are detected in d10 IgG isolated samples in comparison to d10 concentrated supernatants. A and B, Background corrected MFI values for positively assigned HLA class I. C and D, HLA class II antibodies detected in d10 concentrated and d10 IgG isolated culture supernatants vs serum samples (n = 11 [re-tx n = 9 and multiparous n = 2]). MFI, mean fluorescence intensity; re-tx, repeat transplantation.

In 4 (57%) of 7 individuals with HLA class I antibodies in serum, we found HLA class I antibodies in both concentrated and IgG isolated culture supernatants. While there were a total of 126 HLA class I antibody specificities detected in 4 serum samples (median MFI, 3654; range, 419–18 310), a total of 55 HLA class I antibodies were detected in concentrated culture supernatants (median MFI, 1269; range, 12–8706) (Figure 4A) and 82 in IgG isolated samples (median MFI, 4444; range, 248–19 674) (Figure 4B). Among 7 of 9 individuals with serum HLA class II antibodies covering 112 specificities (median, 6218; range, 621–21 233), 5 (56%) individuals had a total of 33 HLA class II-specific antibodies (median, 4201; range, 123–9684) in concentrated culture supernatants (Figure 4C) and 7 (78%) individuals had a total of 66 antibodies in IgG isolated samples (median, 2428; range, 537–20 439) (Figure 4D and **Table S1, SDC,** http://links.lww.com/TP/B653).

Overall, in individuals with serum HLA antibodies, 64% were found to have HLA-specific B-cell memory in concentrated supernatants, whereas 82% showed HLA-specific B–cell memory when IgG isolated supernatants were used for HLA antibody detection.

### HLA Antibody Specificities From Memory B Cells Are Not Identical to Serum HLA Antibody Profiles

**FIGURE 5.**
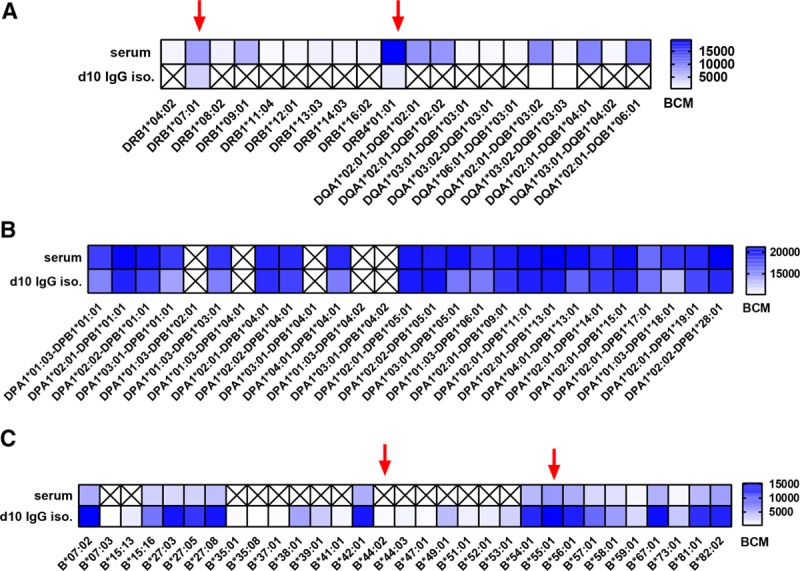
Examples of HLA antibody specificity patterns in paired serum and culture supernatants. A, Limited number of HLA antibody specificities in culture supernatants when compared to serum HLA antibody profiles (Mismatched HLA: DR7/DR53). B, Complete overlap of HLA antibody specificities in serum and supernatant samples (Mismatched HLA-DP unknown). C, Broader specificity of HLA antibodies detected in culture supernatants when compared to serum HLA antibodies (mismatched HLA: B*44:02 and B*55:01). Red arrows indicate mismatched donor/partner HLA. BCM, background corrected mean fluorescence intensity; d10 iso., day 10 IgG isolated; X, no HLA antibody detected.

Having shown that d10 IgG isolated culture supernatants maximize HLA antibody detection by luminex SAB assay, we next compared HLA antibody profiles in these culture supernatants with serum HLA antibody specificities to determine whether memory B cell–derived HLA antibody specificities differed from those found in serum (examples in Figure 5).

A total of 150 HLA class I antibody specificities were detected in serum and d10 IgG isolated samples. While 39% of them were present both in serum and culture supernatants, 45% were present only in serum and 16% were detected only in culture supernatants. For class II, a total of 117 specificities were detected with 53% overlapping in serum and supernatants, whereas 43% detected only in serum and 4% only in culture supernatants.

### No B–cell Memory Detectable in Individuals Without History of Alloantigen Exposure

To determine the specificity of the assay, we tested d10 IgG isolated culture supernatants of individuals without any history of alloantigen exposure (n = 5), for the presence of HLA antibodies using luminex SAB assay. No HLA antibodies were found in supernatants from individuals without a history of alloantigen exposure, despite high IgG concentrations achieved by IgG isolation from d10 culture supernatants (median, 4182 μg/mL; range, 3381–6130 μg/mL; **Figure S4, SDC,** http://links.lww.com/TP/B653). These data confirm that HLA antibodies detected in the d10 IgG isolated culture supernatants of alloantigen-exposed individuals were produced by activated HLA-specific memory B cells.

### Luminex SAB Detected Antibodies in Supernatants Are Directed Against Naturally Expressed HLA Molecules

To determine whether antibodies detected in d10 culture supernatants from alloantigen-exposed individuals were capable of binding cells expressing the corresponding HLA molecules, we performed FCXMs for 3 of the d10 IgG isolated culture supernatants with class I and/or class II HLA antibodies. In accordance with the presence of HLA class I antibodies, 2 samples (AS24 and AS26) were found to be positive for both T and B cells, whereas 1 sample (AS25) with only class II HLA antibodies was positive for B-cell crossmatch in the absence of a positive T-cell crossmatch (Table 1). When the same donor cells were tested with d10 IgG isolated culture supernatants without any HLA antibodies, T- and B-cell flow cytometry crossmatches were negative. These results assured that antibodies detected in d10 IgG isolated samples were truly capable of binding the corresponding HLA molecules present on cells.

**TABLE 1.**
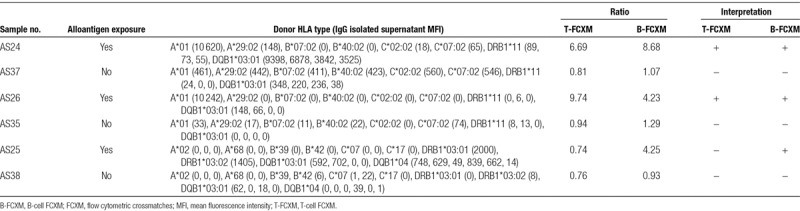
FCXM results of d10 IgG isolated culture supernatants from individuals with and without history of alloantigen exposure

## DISCUSSION

The addition of B-cell supernatant analysis to serum HLA antibody analysis may provide a more complete picture of the potential humoral alloimmune response in transplant recipients with a history of alloantigen exposure. Ideally, to accurately compare HLA antibody specificities detected in serum and those detected in culture supernatants deriving from reactivation of memory B cells, similar sensitivities for HLA antibody detection in both sample sources should be achieved.

Currently available methods to detect HLA-specific memory B cells include tetramer-staining by flow cytometry,^8,13^ ELISPOT-based assays,^9,11,12,18^ and supernatant analysis of B-cell cultures.^7^ Among these, tetramer staining shows only the percentage of B cells binding to certain HLA class I tetramers regardless of their capacity to produce HLA antibodies. Alternatively, HLA-ELISPOT assays allow for detection and quantification of both HLA class I- and class II-specific memory B cells after an in vitro polyclonal activation of peripheral blood B cells. HLA-ELISPOT, although being the only assay enabling quantification of HLA-antibody producing cells, is time consuming and difficult to standardize. On the contrary, testing culture supernatants of activated B cells is an easy and rapid method to detect HLA antibodies with the additional benefit of direct comparison of HLA specificities of the memory B cell and the serum antibody compartment.

Until now, only 2 studies compared HLA antibody specificities detected in culture supernatants and serum of transplant recipients using luminex SAB assays.^7,14^ In both of these studies, neat culture supernatants were 10-fold concentrated using ultra centrifugal filters to increase detectability for HLA antibody screening. In the study of Han et al,^7^ B cells were isolated from PBMC samples before 8 to 14 days of polyclonal stimulation yielding B-cell memory profiles covering 50 specificities in 13 (81%) of 16 of transplant recipients. Although the majority (70%) of DSA found in culture supernatants overlapped with those found in serum, 8% of the DSA were found solely in culture supernatants. Subsequently, Snanoudj et al,^14^ using a polyclonal activation of PBMC for 10 days, found HLA–specific antibodies in culture supernatants of 18 (46%) of 39 patients awaiting a transplant who had serum HLA antibodies. Similar to the study of Han et al, HLA antibody patterns in culture supernatants were not identical to serum HLA antibody profiles.

In the current study, we found increased detectability of HLA antibodies in culture supernatants of which IgG was isolated compared to those that were only 10–fold concentrated, implying an increased sensitivity of HLA-specific B-cell memory detection. Similar to previous studies, we found differences in HLA antibody profiles of serum and culture supernatants of the same individuals. Noteworthy, 16% of HLA class I antibodies and 4% of class II antibodies detected were present only in culture supernatants without corresponding serum specificities, suggesting that HLA-specific memory can be present in the absence of serum HLA antibodies. This finding is particularly important because it supports the notion that some individuals may be at higher risk for de novo antibody formation deriving from activated memory B cells after transplantation. Indeed, re-tx candidates, patients undergoing desensitization treatment or women receiving grafts from their spouse or children comprise a high-risk patient group for developing antibody-mediated rejection because they may harbor HLA-specific memory B cells which can differentiate into antibody producing plasma cells upon re-challenge. Specificity of the current method was confirmed by the lack of HLA-specific memory B cell–derived antibodies in d10 IgG-isolated culture supernatants of individuals without a history of alloantigen exposure.

From previous data on polyclonal B-cell activation systems, we know that IgM can be produced in higher levels than IgG in culture supernatants, likely due to the relatively high percentages of naïve B cells in peripheral blood samples.^19,20^ This can be of importance, because IgM interference in SAB assays has been described previously.^21^ Therefore, the current method to profile HLA antibody specificities in B-cell culture supernatants by isolating and concentrating IgG may eliminate such an interference, if any, and enables a highly sensitive comparison of HLA antibody specificities produced by memory B cells to those found in serum samples.

Inherent to all methods to detect antigen-specific memory B-cell sampling in the peripheral blood compartment, low precursor frequencies, and/or inability to respond to polyclonal activation with R848 may result in a negative test result. Despite the increase in the sensitivity of the assay by isolating and concentrating the total IgG fraction of the culture supernatants, low-frequency HLA-specific memory B cells might still be missed from the peripheral blood compartment. Therefore, a negative result should in our opinion always be reported in relation to the number of B cells tested. Interestingly, our preliminary data on HLA antibody specificity comparison between serum and memory B-cell compartment indicates that these compartments are not necessarily showing similar specificities, which may be of clinical relevance. In this regard, comparative epitope analysis between memory B cell–derived HLA antibodies and serum specificities is warranted in future research. The true clinical relevance of HLA antibodies detected in IgG isolated culture supernatants needs to be established in further clinical studies, which is easily achievable with the current method. Furthermore, clinical studies aiming at longitudinal monitoring of HLA-specific memory B-cell compartment before and different times after transplantation can provide a better understanding on the evolution of preexisting versus de novo developed HLA-specific B-cell memory. This easy to apply method can be efficiently reproduced in other HLA laboratories and has the potential to facilitate further research on the significance of HLA-specific memory B cells in solid organ transplantation.

## ACKNOWLEDGMENTS

The authors thank Jos Drabbels for technical assistance in HLA typing by NGS.

## Supplementary Material
