# Supplementary material for: An Easy and Sensitive Method to Profile the Antibody Specificities of HLA–specific Memory B Cells
Source: Transplantation. 2019 Mar 22;103(4):716–23. doi: 10.1097/TP.0000000000002516 (PMC6430593; doi:10.1097/TP.0000000000002516)
Supplement: Supplementary file 1 [file tp-103-716-s001.pdf]

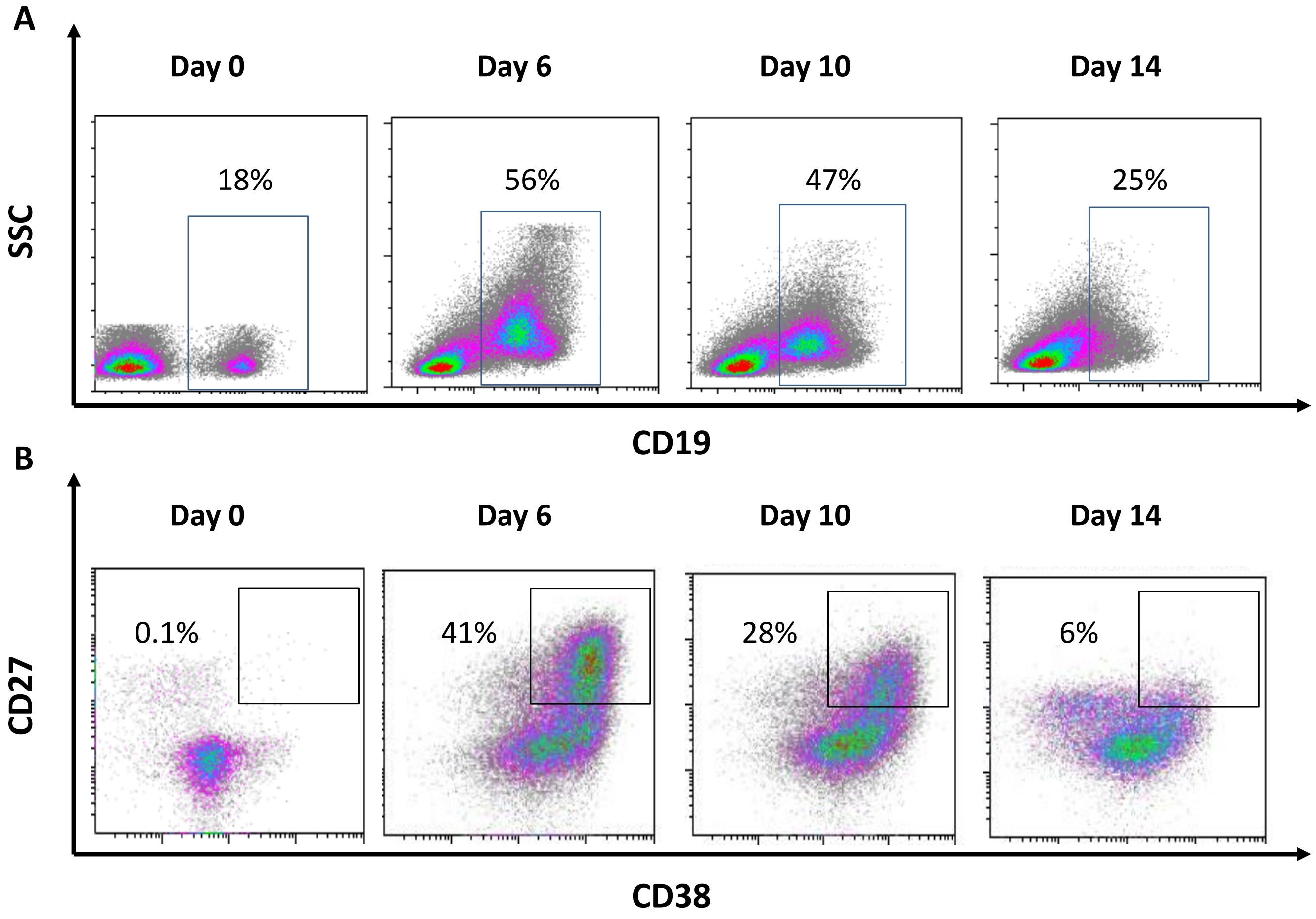

Figure S1: Representative flow cytometry plots before and after polyclonal B cell stimulation. **A)** Percentage of $CD19^{+}$ B cells within the live lymphocyte gate and **B)** Percentage of $CD19^{+}CD27^{+}CD38^{high}$ cells increase significantly from day 0 to day 6 of polyclonal activation, followed by a decrease at day 10 and day 14. (n=9 [re-tx n=3, multi-parous n=1 and alloantigen non-exposed, n=5]). re-tx: repeat transplantation.

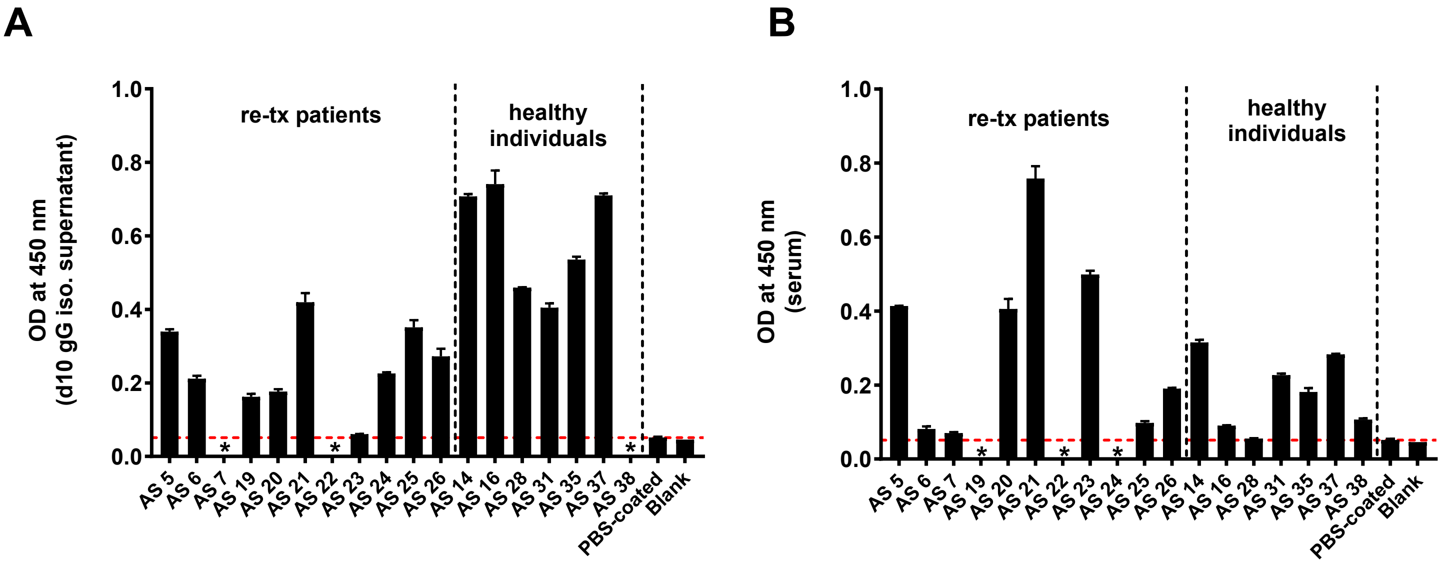

Figure S2: Tetanus toxoid-specific response in (A) d10 IgG isolated culture supernatants and (B) serum samples. To measure the TT-specific IgG responses, ELISA plates were coated with 10 Lf/ml TT antigen diluted in PBS (Dutch Vaccine Institute, Bilthoven, the Netherlands), incubated overnight and were blocked with 2% bovine serum albumin (BSA) in 0.025%Tween-20 in phosphate-buffered saline (PBS-T). Fifty μl of d10 IgG isolated supernatants (1:250 diluted) or serum samples (1:1000 diluted) were incubated for 1h at 37°C. After washing with PBS-T, biotinylated goat anti-human IgG (Novex Life Technologies) was added for 1h at 37°C. Following washing, plates were incubated with streptavidin conjugated to horse radish peroxidase (Thermo-Scientific) for 1h at 37°C. A color reaction was obtained by addition of 4.6 mM 2,2'-azine-bis(3-ethylbenzthiazoline-6-sulphonic acid) (ABTS, Sigma) in a citric acid/PBS buffer at pH 4.2 upon extensive washing. Color reaction was stopped by addition of 250 mM oxalic acid. Optic densitiy (OD) values were obtained by measuring plates at 450 nm. Wells coated only with PBS and wells coated with TT antigen but lacking incubation with the sample (blank) were regarded as negative controls. Results are expressed as mean OD values ± SD of duplicate wells. *Not tested due to insufficient sample material.

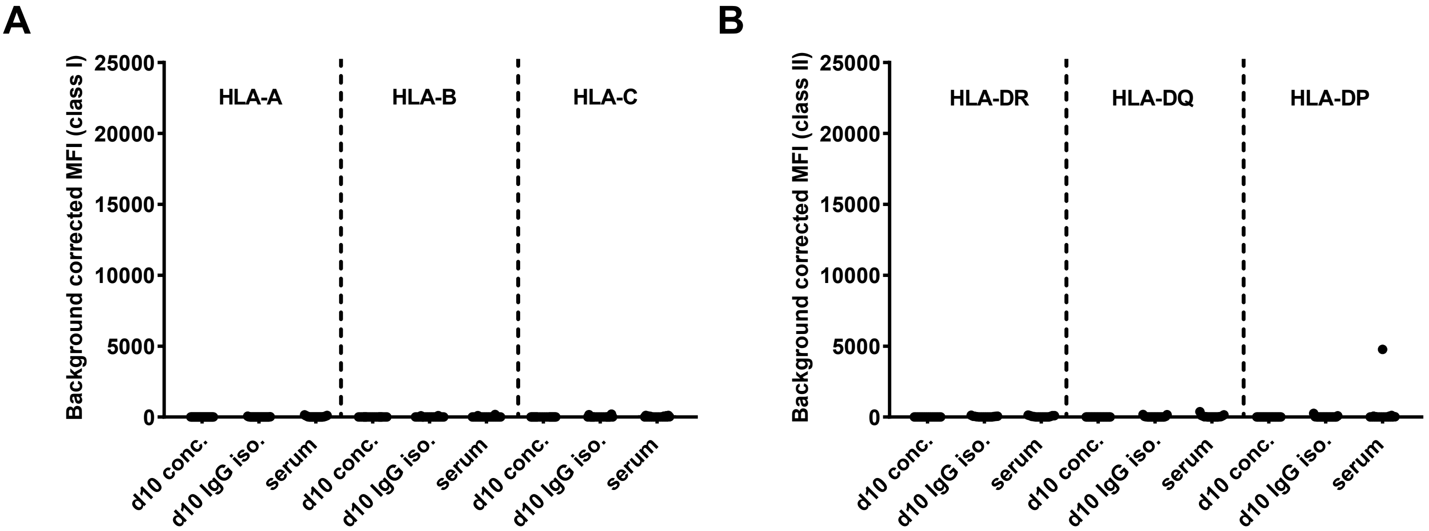

Figure S3: MFI values of self-HLA coated beads. **A)** Background corrected MFI values of self HLA class I and **B)** HLA class II-coated beads measured in d10 concentrated, d10 IgG isolated supernatants and paired serum samples (n=11 [re-tx n=9 and multi-parous n=2]). MFI: Mean fluorescence intensity, re-tx: repeat transplantation, d10 conc.: day 10 concentrated, d10 IgG iso.: day 10 IgG isolated.

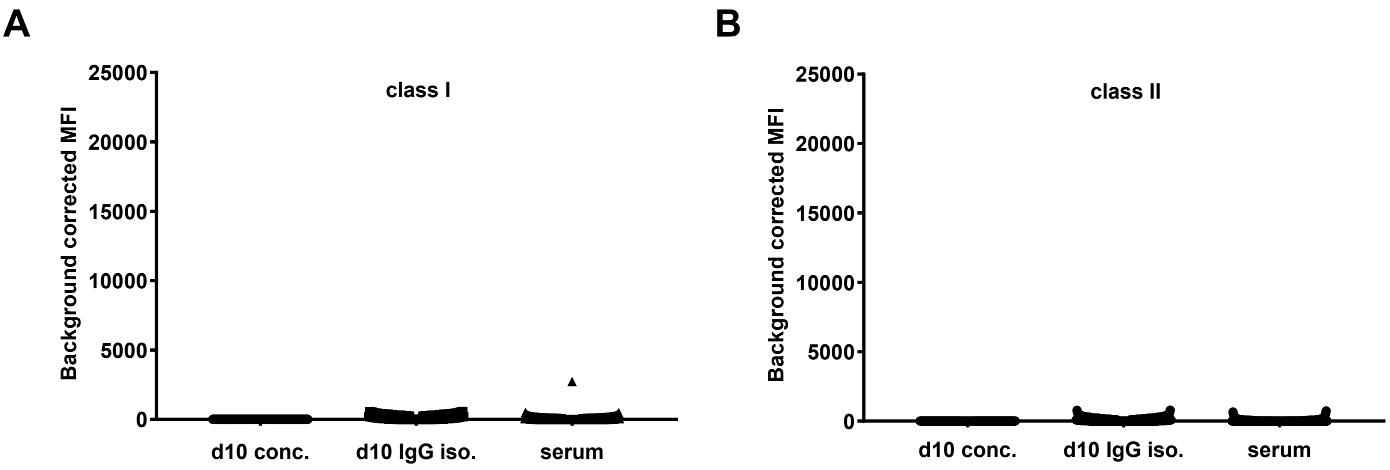

Figure S4: MFI values of samples from individuals without alloantigen exposure. A) Background corrected MFI values of HLA class I and **B)** HLA class II-coated beads measured in d10 concentrated, d10 IgG isolated supernatants and paired serum samples (n=5). MFI: Mean fluorescence intensity, d10 conc.: day 10 concentrated, d10 IgG iso.: day 10 IgG isolated.

Table S1: Immunological overview of alloantigen exposed individuals of the study cohort

**Study code**	AS 5
**Sex**	F
**Study group**	re-tx
**Time (months)***	53
**Self HLA class I**	A*02:01:01, A*03:01:01:01, B*07:02:01, B*40:01:02, C*03:04:01:01, C*07:02:01:03
**Self HLA class II**	DRB1*04:07:01, DRB1*13:02:01, DRB3*03:01:01, DRB4*01:03:01:01, DQB1*03:01:01:01, DQB1*06:04:01, DQA1*01:02:01, DQA1*03, DPB1*03:01:01, DPA1*01:03:01:03, DPA1*01:03:01:03
**Donor (D)/Partner (P)/Child (C) HLA**	**D:** A2, A3, B7, **B44**, **Cw2**, Cw7, DR4, DR13, DQ6, DQ7
**Serum positive class I SABs**	A*23:01, A*24:02, A*24:03, B*13:02, B*14:01, B*14:02, B*15:01, B*15:02, B*15:03, B*15:12, B*15:13, B*15:16, B*15:18, B*18:01, B*27:03, B*27:05, B*27:08, B*35:01, B*35:08, B*37:01, B*38:01, B*39:01, B*44:02, B*44:03, B*45:01, B*46:01, B*47:01, B*49:01, B*50:01, B*51:01, B*52:01, B*53:01, B*54:01, B*55:01, B*56:01, B*57:01, B*58:01, B*59:01, B*67:01, B*78:01, B*82:02, C*02:02, C*04:03, C*05:01, C*06:02, C*15:02, C*17:01, C*18:01
**Supernatant positive class I SABs**	**A*01:01,** A*23:01, A*24:02, B*15:12, B*44:02, B*44:03, B*45:01, B*46:01, B*82:02
**Serum positive class II SABs**	DPA1*01:03-DPB1*02:01, DPA1*01:03-DPB1*04:01, DPA1*02:01-DPB1*04:01, DPA1*02:02-DPB1*04:01, DPA1*03:01-DPB1*04:01, DPA1*04:01-DPB1*04:01, DPA1*01:03-DPB1*04:02, DPA1*03:01-DPB1*04:02, DPA1*02:01-DPB1*15:01, DPA1*01:03-DPB1*18:01, DPA1*02:02-DPB1*28:01
**Supernatant positive class II SABs**	**DRB1*11:01, DRB1*11:03, DRB1*11:04,** DPA1*01:03-DPB1*02:01, DPA1*01:03-DPB1*04:02, DPA1*03:01-DPB1*04:02, DPA1*01:03-DPB1*18:01, DPA1*02:02-DPB1*28:01

Specificities marked with red were found only in supernatants in the absence of serum specificities.
HLA mismatches are marked with bold letters within the available resolution of the HLA typing.
*Time from the last graft failure/delivery to the sampling date.
SABs: Single antigen beads, re-tx: repeat transplantation.

Table S1 continued

**Study code**	AS 6
**Sex**	M
**Study group**	re-tx
**Time (months)***	2
**Self HLA class I**	A*02:01:01, A*32:01:01:01, B*07:02:01, B*40:01:02, C*03:04:01:01, C*07:02:01:03
**Self HLA class II**	DRB1*13:02:01, DRB1*15:01:01, DRB3*03:01:01, DRB5*01, DQB1*06:02:01:01, DQB1*06:04:01, DQA1*01:02:01,<br>DPB1*02:01:02, DPB1*04:02:01:02, DPA1*01:03:01, DPA1*01:03:01:01
**Donor (D)/Partner (P)/Child (C) HLA**	**D1:** A*02:01, **A*31:01**, B*07:02, B*40:01, C*03:04, C*07:02, **DRB1*13:02**, DRB1*15, DQB1*06:02, DQB1*06:04<br>**D2:** A*02:01, A*02:01, **B*13:02**, B*40:01, C*03:04, **C*06:02, DRB1*04:01, DRB1*08:01, DQB1*03:01, DQB1*04:02**
**Serum positive class I SABs**	A*29:01, A*29:02, A33:01
**Supernatant positive class I SABs**	negative
**Serum positive class II SABs**	DRB1*04:01, DRB1*04:02, DRB1*04:03, DRB1*04:04, DRB1*04:05,<br>DQA1*03:01-DQB1*03:01, DQA1*03:02-DQB1*03:01, DQA1*05:01-DQB1*03:01, DQA1*06:01-DQB1*03:01, DQA1*02:01-DQB1*03:02, DQA1*03:01-DQB1*03:02, DQA1*03:02-DQB1*03:02, DQA1*03:02-DQB1*03:03, DQA1*04:01-DQB1*03:03, DQA1*06:01-DQB1*03:03, DQA1*02:01-DQB1*04:01, DQA1*04:01-DQB1*04:01, DQA1*05:01-DQB1*04:01, DQA1*03:01-DQB1*04:02, DQA1*04:01-DQB1*04:02, DQA1*06:01-DQB1*04:02
**Supernatant positive class II SABs**	negative

Table S1 continued

**Study code**	AS 7
**Sex**	F
**Study group**	re-tx
**Time (months)***	27
**Self HLA class I**	A*01:01:01:01, A*03:01:01:01, B*08:01:01:01, B*15:01:01:03, C*03:04:01:01, C*07:01:01:01
**Self HLA class II**	DRB1*04:08:01, DRB1*13:01:01, DRB3*02:02:01:02, DRB4*01:03:01:01, DQB1*03:04:01, DQB1*06:03:01, DQA1*01:03:01, DQA1*03:03:01:01, DPB1*04:01:01, DPB1*15:01:01, DPA1*01:03:01:02, DPA1*01:04
**Donor (D)/Partner (P) /Child (C) HLA**	**D:** A*01:01, **A*68:01,** B*08:01, **B*18:01**, C*07:01, **DRB1*04:03**, DRB1*13:01, **DQB1*03:02**, DQB1*06:03 **P: A*26:01, A*68:01**, **B*44:02**, **B*55:01**, **C*03:03, C*07:04,** DRB1*13:01, **DRB1*14:54**, DQB1*05:03 **DQB1*06:04**
**Serum positive class I SABs**	A*02:01, A*02:02, A*02:03, A*02:05, A*11:01, A*11:02, A*23:01, A*24:02, A*24:03, A*25:01, A*26:01, A*33:01, A*33:03, A*34:02, A*43:01, A*66:01, A*66:02, A*68:01, A*68:02, A*69:01, B*07:02, B*15:16, B*27:03, B*27:05, B*27:08, B*42:01, B*54:01, B*55:01, B*56:01, B*57:01, B*58:01, B*59:01, B*67:01, B*73:01, B*81:01, B*82:02
**Supernatant positive class I SABs**	A*02:01, A*02:02, A*02:03, A*02:05, A*11:01, A*11:02, A*24:02, A*24:03, A*25:01, A*26:01, **A*29:01**, **A*29:02**, **A*32:01**, A*33:01, A*33:03, A*34:02, **A*36:01**, A*43:01, A*66:01, A*66:02, A*68:01, A*68:02, A*69:01, **A*74:01**, B*07:02, **B*07:03**, **B*15:13**, B*15:16, B*27:03, B*27:05, B*27:08, **B*35:01**, **B*35:08, B*37:01, B*38:01**, **B*39:01, B*41:01**, B*42:01, **B*44:02, B*44:03, B*47:01, B*49:01, B*51:01, B*52:01, B*53:01**, B*54:01, B*55:01, B*56:01, B*57:01, B*58:01, B*59:01, B*67:01, B*73:01, B*81:01, B*82:02
**Serum positive class II SABs**	negative
**Supernatant positive class II SABs**	negative

Table S1 continued

**Study code**	AS 14
**Sex**	F
**Study group**	multi-parous
**Time (months)***	108
**Self HLA class I**	A*02:01:01, A*24:02:01:01, B*07:02:01, C*07:02:01:03
**Self HLA class II**	DRB1*04:04:01, DRB1*15:01:01, DRB4*01:03:01:01, DRB5*01:01:01, DQB1*03:02:01, DQB1*06:02:01:01, DQA1*01:02:01, DQA1*03:01:01, DPB1*04:01:01, DPA1*01:03:01:04
**Donor (D)/Partner (P)/Child (C) HLA**	**C:** A*02:01, A*24, B*07, **B*40:01,** C*03:04, C*07:02, DRB1*04:04, DRB1*15:01, DRB4, DRB5, DQB1*03:02, DQB1*06:02
**Serum positive class I SABs**	B*13:02, B*14:01, B*15:01, B*15:02, B*15:03, B*15:12, B*15:13, B*15:18, B*18:01, B*35:01, B*35:08, B*37:01, B*38:01, B*39:01, B*40:01, B*40:02, B*41:01, B*44:02, B*44:03, B*45:01, B*47:01, B*48:01, B*49:01, B*50:01, B*51:01, B*52:01, B*53:01, B*54:01, B*55:01, B*56:01, B*59:01, B*78:01
**Supernatant positive class I SABs**	B*13:02, B*15:01, B*15:12, **B*27:05, B*27:08**, B*40:01, B*40:02, B*41:01, B*44:02, B*44:03, B*45:01, B*47:01, B*49:01, B*50:01
**Serum positive class II SABs**	negative
**Supernatant positive class II SABs**	negative

Table S1 continued

**Study code**	AS 16
**Sex**	F
**Study group**	multi-parous
**Time (months)***	346
**Self HLA class I**	A*11:01:01:01, A*25:01:01, B*15:01:01:01, B*18:01:01, C*03:03:01:01, C*12:03:01:01
**Self HLA class II**	DRB1*08:01/08*77, DRB1*13:01:01, DRB3*02:02:01:02, DQB1*04:02:01, DQB1*06:03:01, DQA1*01:03/01:10/01:14, DQA1*04:01/04:02/04:03N, DPB1*02:01, DPB1*04:01:01, DPA1*01:03:01
**Donor (D)/Partner (P)/Child (C) HLA**	**P: A*02, A*02, B*39, B*40**, C*03, C*07, DRB1*08, DRB1*13, DRB3, DQB1*04, DQB1*06, **DQA1*01:02**, DQA1*04:01
**Serum positive class I SABs**	negative
**Supernatant positive class I SABs**	negative
**Serum positive class II SABs**	DQA1*03:01-DQB1*03:02, DQA1*03:02-DQB1*03:02
**Supernatant positive class II SABs**	**DQA1*03:01-DQB1*03:01**, DQA1*03:01-DQB1*03:02, DQA1*03:02-DQB1*03:02, **DQA1*03:02-DQB1*03:03**

Table S1 continued

**Study code**	AS 19
**Sex**	M
**Study group**	re-tx
**Time (months)***	114
**Self HLA class I**	A*01:01:01:01, A*26:01:01:01, B*15:01:01:01, B*38:01:01, C*03:03:01:01, C*12:03:01:01
**Self HLA class II**	DRB1*13:02:01, DRB1*15:01:01, DRB3*03:01:01, DRB5*01:01:01, DQB1*06:02:01:01, DQB1*06:09:01:01, DQA1*01:02:01, DPB1*04:01:01, DPA1*01:03:01:04
**Donor (D)/Partner (P)/Child (C) HLA**	**D:** A1, **A11, B8**, B62, Cw3, **DR3**, DR15, DQ1, **DQ2**
**Serum positive class I SABs**	not available
**Supernatant positive class I SABs**	negative
**Serum positive class II SABs**	not available
**Supernatant positive class II SABs**	DQA1*05:01-DQB1*02:01, DQA1*03:02-DQB1*02:02, DQA1*05:01-DQB1*02:02, DQA1*03:01-DQB1*03:01, DQA1*03:02-DQB1*03:01, DQA1*05:01-DQB1*03:01, DQA1*06:01-DQB1*03:01, DQA1*03:01-DQB1*03:02, DQA1*03:02-DQB1*03:02, DQA1*03:02-DQB1*03:03, DQA1*04:01-DQB1*03:03, DQA1*06:01-DQB1*03:03, DQA1*04:01-DQB1*04:01, DQA1*05:01-DQB1*04:01, DQA1*04:01-DQB1*04:02, DQA1*06:01-DQB1*04:02

Table S1 continued

**Study code**	AS 20
**Sex**	F
**Study group**	re-tx
**Time (months)***	74
**Self HLA class I**	A*23:01:01:01, A*31:01:02:01, B*40:01:02, B*50:02, C*03:04:01:01, C*06:02:01:02
**Self HLA class II**	DRB1*04:04:01, DRB1*04:06:02, DRB4*01:03:01:01, DQB1*03:02:01, DQB1*04:02:01, DQA1*03:01:01, DQA1*03:03:01:01, DPB1*03:01/124:01, DPB1*04:01/350:01, DPA1*01:03:01:02, DPA1*01:03:01:03
**Donor (D)/Partner (P)/Child (C) HLA**	**D: A1, A24, B8**, B60, Cw3, **Cw7, DR1**, DR4, **DQ1**, DQ3
**Serum positive class I SABs**	A*01:01, B*08:01, B*35:01, B*35:08, B*37:01, B*42:01, B*51:01, B*53:01, B*59:01
**Supernatant positive class I SABs**	negative
**Serum positive class II SABs**	DRB1*01:01, DRB1*01:02, DRB1*01:03, DRB1*04:02, DRB1*08:01, DRB1*08:02, DRB1*11:01, DRB1*11:03, DRB1*11:04, DRB1*12:01, DRB1*12:02, DRB1*13:01, DRB1*13:03, DRB1*13:05, DRB1*14:03, DRB1*16:01, DRB1*16:02, DRB5*01:01, DRB5*02:02, DQA1*01:01-DQB1*05:01, DQA1*01:02-DQB1*05:01, DQA1*01:02-DQB1*05:02, DQA1*01:04-DQB1*05:03, DQA1*01:03-DQB1*06:01, DQA1*01:04-DQB1*06:01, DQA1*02:01-DQB1*06:01, DQA1*01:02-DQB1*06:02, DQA1*01:03-DQB1*06:03, DQA1*01:02-DQB1*06:04
**Supernatant positive class II SABs**	DQA1*01:01-DQB1*05:01, DQA1*01:02-DQB1*05:01, DQA1*01:02-DQB1*05:02, DQA1*01:04-DQB1*05:03, DQA1*01:03-DQB1*06:01, DQA1*01:04-DQB1*06:01, DQA1*02:01-DQB1*06:01, DQA1*01:02-DQB1*06:02, DQA1*01:03-DQB1*06:03, DQA1*01:02-DQB1*06:04

Table S1 continued

**Study code**	AS 21
**Sex**	F
**Study group**	re-tx
**Time (months)***	72
**Self HLA class I**	A*02:01:01, A*29:02:01:01, B*44:03:01, B*73:01, C*15:05:01, C*16:01:01:01
**Self HLA class II**	DRB1*04:05:01, DRB1*13:02:01, DRB3*03:01:01, DRB4*01:03:01:01, DQB1*02:02:01:02, DQB1*06:04:01, DQA1*01:02:01, DQA1*03:03:01:01, DPB1*02:01:02, DPB1*13:01/107:01, DPA1*01:03:01:01, DPA1*02:01:01:01
**Donor (D)/Partner (P)/Child (C) HLA**	**D1:** unknown<br>**D2:** A2, B44, **B50**, DR4, DR13, **DQ7**<br>**D3:** A2, B44, DR4, DR13, DQ1, **DQ3**
**Serum positive class I SABs**	negative
**Supernatant positive class I SABs**	negative
**Serum positive class II SABs**	DQA1*03:01-DQB1*03:01, DQA1*03:02-DQB1*03:01, DQA1*05:01-DQB1*03:01, DQA1*06:01-DQB1*03:01, DQA1*02:01-DQB1*03:02, DQA1*03:01-DQB1*03:02, DQA1*03:02-DQB1*03:02, DQA1*03:02-DQB1*03:03, DQA1*04:01-DQB1*03:03, DQA1*06:01-DQB1*03:03
**Supernatant positive class II SABs**	DQA1*02:01-DQB1*03:02, DQA1*03:01-DQB1*03:02, DQA1*03:02-DQB1*03:02, DQA1*03:02-DQB1*03:03, DQA1*04:01-DQB1*03:03

Table S1 continued

**Study code**	AS 22
**Sex**	M
**Study group**	re-tx
**Time (months)***	132
**Self HLA class I**	A*01:01:01:01, A*02:01:01, B*08:01:01:01, B*15:01:01:01, C*03:04:01:01, C*07:01:01:01
**Self HLA class II**	DRB1*03:01:01, DRB1*04:01:01:02, DRB3*01:01:02, DRB4*01:03:01:01, DQB1*02:01:01, DQB1*03:02:01, DQA1*03:01:01, DQA1*05:01:01:02, DPB1*04:01:01, DPA1*01:03:01:02, DPA1*01:03:01:04
**Donor (D)/Partner (P)/Child (C) HLA**	**D:** A2, **A11**, B8, B62, DR17, DR4, DQ2, DQ8
**Serum positive class I SABs**	negative
**Supernatant positive class I SABs**	negative
**Serum positive class II SABs**	DPA1*01:03-DPB1*01:01, DPA1*02:01-DPB1*01:01, DPA1*02:02-DPB1*01:01, DPA1*03:01-DPB1*01:01, DPA1*01:03-DPB1*03:01, DPA1*02:01-DPB1*04:01, DPA1*02:02-DPB1*04:01, DPA1*04:01-DPB1*04:01, DPA1*02:01-DPB1*05:01, DPA1*02:02-DPB1*05:01, DPA1*03:01-DPB1*05:01, DPA1*01:03-DPB1*06:01, DPA1*02:01-DPB1*09:01, DPA1*02:01-DPB1*11:01, DPA1*02:01-DPB1*13:01, DPA1*04:01-DPB1*13:01, DPA1*02:01-DPB1*14:01, DPA1*02:01-DPB1*15:01, DPA1*02:01-DPB1*17:01, DPA1*01:03-DPB1*18:01, DPA1*02:01-DPB1*19:01, DPA1*02:02-DPB1*28:01
**Supernatant positive class II SABs**	DPA1*01:03-DPB1*01:01, DPA1*02:01-DPB1*01:01, DPA1*02:02-DPB1*01:01, DPA1*03:01-DPB1*01:01, DPA1*01:03-DPB1*03:01, DPA1*02:01-DPB1*04:01, DPA1*02:02-DPB1*04:01, DPA1*04:01-DPB1*04:01, DPA1*02:01-DPB1*05:01, DPA1*02:02-DPB1*05:01, DPA1*03:01-DPB1*05:01, DPA1*01:03-DPB1*06:01, DPA1*02:01-DPB1*09:01, DPA1*02:01-DPB1*11:01, DPA1*02:01-DPB1*13:01, DPA1*04:01-DPB1*13:01, DPA1*02:01-DPB1*14:01, DPA1*02:01-DPB1*15:01, DPA1*02:01-DPB1*17:01, DPA1*01:03-DPB1*18:01, DPA1*02:01-DPB1*19:01, DPA1*02:02-DPB1*28:01

Table S1 continued

**Study code**	AS 23
**Sex**	F
**Study group**	re-tx
**Time (months)***	2
**Self HLA class I**	A*01:01:01:01, A*02:01:01, B*08:01:01:01, B*40:01:02, C*03:04:01:01, C*07:01:01:01
**Self HLA class II**	DRB1*03:01:01, DRB1*13:02:01, DRB3*01:01:02, DRB3*03:01:01, DQB1*02:01:01, DQB1*06:04:01, DQA1*01:02:01, DQA1*05:01:01:02, DPB1*03:01/124:01, DPB1*04:01/350:01, DPA1*01:03:01:02, DPA1*01:03:01:03
**Donor (D)/Partner (P)/Child (C) HLA**	D1: A1, A2, B8, B15<br>D2: A1, **A24,** B8, **B41**, Cw7, DR3, DR13, DQ2, **DQ7**
**Serum positive class I SABs**	negative
**Supernatant positive class I SABs**	negative
**Serum positive class II SABs**	DRB1*04:04, DRB1*04:05, DRB4*01:01, DQA1*03:02-DQB1*02:02,DQA1*03:01-DQB1*03:01, DQA1*03:02-DQB1*03:01, DQA1*05:01-DQB1*03:01, DQA1*06:01-DQB1*03:01, DQA1*02:01-DQB1*03:02, DQA1*03:01-DQB1*03:02, DQA1*03:02-DQB1*03:02, DQA1*03:02-DQB1*03:03, DQA1*04:01-DQB1*03:03, DQA1*06:01-DQB1*03:03, DQA1*03:01-DQB1*04:02
**Supernatant positive class II SABs**	negative

Table S1 continued

**Study code**	AS 24
**Sex**	F
**Study group**	re-tx
**Time (months)***	3
**Self HLA class I**	A*02:01:01, B*08:01:01:01, B*18:01:01, C*07:01:01:01, C*12:03:01:01
**Self HLA class II**	DRB1*15:01:01, DRB5*01:01:01, DQB1*06:02:01:01, DQA1*01:02:01, DPB1*01:01:01, DPB1*13:01/107:01, DPA1*02:01:01:01, DPA1*02:02:02
**Donor (D)/Partner (P)/Child (C) HLA**	**D: A1,** A2, B18, **B57**, **Cw6,** Cw12, **DR13**, DR15, DR51, **DR52, DQ3,** DQ6
**Serum positive class I SABs**	not available
**Supernatant positive class I SABs**	A*01:01, A*11:01, A*11:02, A*23:01, A*24:02, A*25:01, A*26:01, A*30:01, A*32:01, A*34:02, A*36:01, A*66:01, A*66:02, B*15:12, B*15:13, B*15:16, B*44:02, B*44:03, B*45:01, B*57:01, B*58:01
**Serum positive class II SABs**	not available
**Supernatant positive class II SABs**	DQA1*05:01-DQB1*02:01, DQA1*03:02-DQB1*02:02, DQA1*05:01-DQB1*02:02, DQA1*03:01-DQB1*03:01, DQA1*03:02-DQB1*03:01, DQA1*05:01-DQB1*03:01, DQA1*06:01-DQB1*03:01, DQA1*02:01-DQB1*03:02, DQA1*03:01-DQB1*03:02, DQA1*03:02-DQB1*03:02, DQA1*03:02-DQB1*03:03, DQA1*04:01-DQB1*03:03, DQA1*06:01-DQB1*03:03, DQA1*04:01-DQB1*04:01, DQA1*05:01-DQB1*04:01, DQA1*03:01-DQB1*04:02, DQA1*04:01-DQB1*04:02, DQA1*06:01-DQB1*04:02

Table S1 continued

**Study code**	AS 25
**Sex**	F
**Study group**	re-tx
**Time (months)***	121
**Self HLA class I**	A*01:01:01:01, B*37:01:01, B*57:01:01, C*06:02:01:01
**Self HLA class II**	DRB1*04:02:01, DRB1*07:01:01, DRB4*01:03:01:01, DRB4*01:03:01:02N, DQB1*03:02:01, DQB1*03:03:02:01, DQA1*02:01:01, DQA1*03:01:01, DPB1*04:01:01, DPB1*13:01/107:01, DPA1*01:03:01:04, DPA1*02:01:01:01
**Donor (D)/Partner (P)/Child (C) HLA**	**D:** A1, **A32,** B17, **B40, Cw3**, Cw6, **DR3,** DR7, **DQ2**
**Serum positive class I SABs**	A*02:01, A*02:02, A*02:03, A*02:05, A*11:02, A*68:01, A*68:02, B*13:02, B*40:01, B*82:02
**Supernatant positive class I SABs**	negative
**Serum positive class II SABs**	DRB1*03:01, DRB1*03:02, DRB1*03:03, DRB3*01:01, DQA1*02:01-DQB1*02:01, DQA1*05:01-DQB1*02:01, DQA1*02:01-DQB1*02:02, DQA1*03:02-DQB1*02:02, DQA1*05:01-DQB1*02:02, DQA1*05:01-DQB1*03:01, DQA1*06:01-DQB1*03:01, DQA1*04:01-DQB1*03:03, DQA1*06:01-DQB1*03:03, DQA1*04:01-DQB1*04:01, DQA1*05:01-DQB1*04:01, DQA1*04:01-DQB1*04:02, DQA1*06:01-DQB1*04:02, DQA1*01:02-DQB1*05:02
**Supernatant positive class II SABs**	DRB1*03:01, DRB1*03:02, DRB1*03:03, DRB3*01:01, DQA1*05:01-DQB1*02:01, DQA1*05:01-DQB1*02:02, DQA1*05:01-DQB1*03:01, DQA1*04:01-DQB1*03:03, DQA1*06:01-DQB1*03:03, DQA1*04:01-DQB1*04:01, DQA1*05:01-DQB1*04:01, DQA1*04:01-DQB1*04:02, DQA1*06:01-DQB1*04:02

Table S1 continued

**Study code**	AS 26
**Sex**	F
**Study group**	re-tx
**Time (months)***	56
**Self HLA class I**	A*11:01:01:01, A*29:02:01:01, B*18:01:01, B*50:01:01:02, C*04:01:01:01, C*05:01:01:01
**Self HLA class II**	DRB1*03:01:01, DRB1*10:01:01, DRB3*02:02:01:01, DQB1*02:01:01, DQB1*05:01:01, DQA1*01:05:01, DQA1*05:01:01:01, DPB1*04:02:01:02, DPB1*30:01, DPA1*01:03:01:02, DPA1*02:01:01:01
**Donor (D)/Partner (P)/Child (C) HLA**	**D: A1, A33,** B18, B50, Cw5, **Cw6**, DR17, **DR7, DR53,** DQ2
**Serum positive class I SABs**	A*01:01, A*23:01, A*24:02, A*36:01, A*80:01, B*15:12, B*44:02, B*44:03, B*45:01, B*82:02
**Supernatant positive class I SABs**	A*01:01, A*24:02, A*36:01, **A*66:02**
**Serum positive class II SABs**	DRB1*04:02, DRB1*07:01, DRB1*08:02, DRB1*09:01, DRB1*11:04, DRB1*12:01, DRB1*13:03, DRB1*14:03, DRB1*16:02, DRB4*01:01, DQA1*02:01-DQB1*02:01, DQA1*02:01-DQB1*02:02, DQA1*03:01-DQB1*03:01, DQA1*03:02-DQB1*03:01, DQA1*06:01-DQB1*03:01, DQA1*02:01-DQB1*03:02, DQA1*03:02-DQB1*03:03, DQA1*02:01-DQB1*04:01, DQA1*03:01-DQB1*04:02, DQA1*02:01-DQB1*06:01
**Supernatant positive class II SABs**	DRB1*07:01, DRB4*01:01, DQA1*02:01-DQB1*03:02, DQA1*03:02-DQB1*03:03
